# Commercial Biocides Induce Transfer of Prophage Φ13 from Human Strains of *Staphylococcus aureus* to Livestock CC398

**DOI:** 10.3389/fmicb.2017.02418

**Published:** 2017-12-07

**Authors:** Yuanyue Tang, Lene N. Nielsen, Annemette Hvitved, Jakob K. Haaber, Christiane Wirtz, Paal S. Andersen, Jesper Larsen, Christiane Wolz, Hanne Ingmer

**Affiliations:** ^1^Department of Veterinary and Animal Science, Faculty of Health and Medical Sciences, University of Copenhagen, Frederiksberg, Denmark; ^2^National Food Institute, Technical University of Denmark, Søborg, Denmark; ^3^DTU Biosustain, Technical University of Denmark, Kongens Lyngby, Denmark; ^4^Institut für Medizinische Mikrobiologie und Hygiene, Universitätsklinikum Tübingen, Tübingen, Germany; ^5^Department of Microbiology and Infection Control, Statens Serum Institut, Copenhagen, Denmark

**Keywords:** LA-MRSA CC398, biocide, prophage, ΦSa3, phage transfer

## Abstract

Human strains of *Staphylococcus aureus* commonly carry the bacteriophage ΦSa3 that encodes immune evasion factors. Recently, this prophage has been found in livestock-associated, methicillin resistant *S. aureus* (MRSA) CC398 strains where it may promote human colonization. Here, we have addressed if exposure to biocidal products induces phage transfer, and find that during co-culture, Φ13 from strain 8325, belonging to ΦSa3 group, is induced and transferred from a human strain to LA-MRSA CC398 when exposed to sub-lethal concentrations of commercial biocides containing hydrogen peroxide. Integration of ΦSa3 in LA-MRSA CC398 occurs at multiple positions and the integration site influences the stability of the prophage. We did not observe integration in *hlb* encoding β-hemolysin that contains the preferred ΦSa3 attachment site in human strains, and we demonstrate that this is due to allelic variation in CC398 strains that disrupts the phage attachment site, but not the expression of β-hemolysin. Our results show that hydrogen peroxide present in biocidal products stimulate transfer of ΦSa3 from human to LA-MRSA CC398 strains and that in these strains prophage stability depends on the integration site. Knowledge of ΦSa3 transfer and stability between human and livestock strains may lead to new intervention measures directed at reducing human infection by LA-MRSA strains.

## Introduction

*Staphylococcus aureus* is an opportunistic pathogen in human and animals, and is one of the leading causes of acute and chronic infections. When treating staphylococcal infections, resistance to β-lactam antibiotics is an increasing problem with methicillin resistant *S. aureus* (MRSA) clones epidemically spreading in the hospitals as well as in the community (DeLeo et al., 2010; Dulon et al., 2011). In recent years, strains belonging to clonal complex 398 (CC398) have been widely detected in pigs and have also been found in other livestock animals such as turkey, chicken and cattle (Smith and Pearson, 2011). In general, livestock-associated MRSA (LA-MRSA) CC398 is regarded less virulent than human-associated MRSA clones due to the absence of several common virulence factors, such as enterotoxins and phage encoded Panton-Valentine leukocidin (PVL) (Schijffelen et al., 2010; Price et al., 2012; Becker et al., 2015). However, LA-MRSA CC398 causes infections in humans with livestock contact (Köck et al., 2011; Pérez-Moreno et al., 2016) and since first detected in the early 2000s (van Loo et al., 2007) the number of clinical cases in humans has increased steadily (van Cleef et al., 2011; Cuny et al., 2015). The infection of humans by LA-MRSA CC398 is primarily regarded as an occupational risk for farmers, veterinarians and meat handlers (Smith and Pearson, 2011) but a secondary risk is the spread of CC398 to the community (Smith and Pearson, 2011; Smith, 2015). In fact, infections of LA-MRSA CC398 in patients without animal contact have been reported both in Europe and the United States (Welinder-Olsson et al., 2008; Wulf et al., 2008; Bhat et al., 2009; Larsen et al., 2015, 2016; Diene et al., 2017).

The ability of *S. aureus* to colonize host organisms is in part determined by prophages. The majority of human-associated *S. aureus* isolates contain β-hemolysin negative-converting bacteriophages, which are classified as ΦSa3 (Goerke et al. 2006b). They share an integrase (Goerke et al., 2009) and an attachment core sequence (5′-TGTATCCAAACTGG-3′) in the *hlb* gene (Coleman et al., 1991). ΦSa3 phages contain the immune evasion cluster (IEC) encoding the chemotaxin inhibitory protein (CHIPS), staphylococcal complement inhibitor (SCIN), staphylokinase (SAK), as well as some enterotoxins (Coleman et al., 1989; van Wamel et al., 2006; Goerke et al., 2009; McCarthy and Lindsay, 2010), which contribute to adaptation to the human host (de Haas et al., 2004; Rooijakkers et al., 2005; Jung et al., 2016). Recent data suggest that ΦSa3 phages are occasionally found in livestock-associated *S. aureus* strains, and the acquisition of ΦSa3 is important for the host-jump of *S. aureus* from animals to humans (Price et al., 2012; van der Mee-Marquet et al., 2014; Larsen et al., 2015, 2016).

Induction of prophages generally occurs in response to DNA damage elicited by for example reactive oxygen species and some antibiotics, and is mediated by RecA activation and cleavage of the phage repressor (Frye et al., 2005; Goerke et al., 2006a; Nanda et al., 2015). Thus, extrinsic factors contribute to the mobility of ΦSa3 phages among *S. aureus* strains. Disinfection and cleaning are critical steps when maintaining a desired hygiene status in household products, in hospitals and in food animal production. In livestock production, biocides are widely applied for cleaning and disinfection of animal-associated production areas, equipment, during transport and even directly on animals to prevent skin diseases (Quinn and Markey, 2001). Major biocides applied in livestock production include hydrogen peroxide, peracetic acid, glutaric aldehyde, quaternary ammonium compounds, and isopropanol (Kjølholt et al., 2001; SCHENIHR (Scientific Committee on Emerging and Newly Identified Health Risks), 2009). Hydrogen peroxide is a common disinfectant applied in animal production. It is a powerful oxidizing agent that oxidizes thiol groups in enzymes and proteins and leads to free radical production (Kjølholt et al., 2001; Russell, 2003). Here we have applied a prophage Φ13 from strain 8325 belonging to ΦSa3 group to examine if sub-lethal concentrations of commercial biocides and biocidal compounds induce phage ΦSa3 and stimulate its transmission to LA-MRSA CC398 strains.

## Materials and methods

### Strain collection and chemical reagents

Chemicals and biocides used in this study are listed in Table 1 and strains in Table 2. Media used in this study included Mueller-Hinton broth (MHB), tryptic soy broth (TSB) and agar (TSA) from Oxoid. Sheep blood is from Department of Veterinary Disease Biology, University of Copenhagen.

**Table 1:** Biocides and chemical regents included in this study.

**Regents**	**Composition**	**Manufacturer**
Biocide 1^*^	Hydrogen peroxide (15–30%) Acetic acid (5–15%) Peracetic acid (1–5%)	Novadan
Biocide 2^*^	Hydrogen peroxide (15–30%) Acetic acid (1–5%) Peracetic acid (1–5%) Phosphonic acid (1–5%) Amine oxide (1–5%)	Novadan
Biocide 3^*^	Active chlorine (60–100%) Sodium hydroxide (1–5%) Amine oxide (<1%)	Novadan
Mitomycin C	Mitomycin C from *Streptomyces* caespitosus	Sigma-Aldrich
Hydrogen peroxide	Hydrogen peroxide (30%)	Sigma-Aldrich
Benzalkonium chloride	Benzalkonium chloride (10%)	Sigma-Aldrich
Acetic acid	Acetic acid (100%)	Merck
Amine oxide	N,N-Dimethyldodecylamine N-oxide (30%)	Sigma-Aldrich
Peracetic acid	Peracetic acid (38–40%)	Merck

* *Represents commercial biocide*.

**Table 2:** Bacteria strains included in this study.

**Strain**	**Description**	**References**
8325-4	NCTC8325 phage-cured	Novick, 1967
8325-4Φ13 (CG1)	8325-4 lysogenized with Φ13	Goerke et al., 2006a
RN4220	Restriction defective derivative of 8325-4	Kreiswirth et al., 1983
8325-4Φ13-kana	8325-4 lysogenized with Φ13 *chips, scin*::*aph*A3, kanamycin resistant	This study
MW2-ΦSa3mw	MW2-ΦSa3mw	Wirtz et al., 2009
MW2c	MW2 phage-cured	This study
61599	*spa* type t034, tetracycline resistant, *hlb*^+^	Larsen et al., 2015
93616	*spa* type t899, tetracycline resistant, *hlb*^+^	Larsen et al., 2016
DC10B	Δ*dcm* in DH10B background; Dam methylation only	Monk et al., 2012
8325-4Φ13attBmut	8325-4 mutated at Φ13 *att*B site in *hlb, hlb*^+^	This study
RN4220Φ13attBmut	RN4220 mutated at Φ13 *att*B site in *hlb, hlb*^+^	This study

### Strain construction

Phage-cured MW2c was obtained by treating MW2-ΦSa3mw (Wirtz et al., 2009) with 1 μg/ml mitomycin C for 2 h at 37°C, 200 rpm in TSB and subsequently with 0.5 mM hydrogen peroxide for 3 h at 37°C, 200 rpm. Serial dilutions were plated on TSA with 0.5 mM hydrogen peroxide and incubated at 37°C for overnight. Colonies grew on the plates were selected as phage cured bacteria (MW2c) and checked by PFGE (Goerke et al., 2004) for lacking of the phage.

Strain 8325-4Φ13-kana was obtained by replacing part of the 3′-end of Φ13 (*chps* and *scin*) in strain Φ8325-4Φ13 (Goerke et al., 2006a) with kanamycin resistance cassette *aph*A3. In brief, two fragments flanking the 3′-region of Φ13 and the kanamycin-resistant cassette from pDG782 (Guérout-Fleury et al., 1995) were amplified and annealed by overlapping PCR. The amplicon was cloned into *Kpn*I restriction site into pBT2 (Brückner, 2006) to gain pCG6, which was electroporated into strain RN4220 (Kreiswirth et al., 1983) and further transduced into 8325-4Φ13. pCG6 was then used to mutagenize strain 8325-4Φ13 as described by Brückner (2006) to obtain strain 8325-4Φ13kana, and confirmed by sequencing.

To identify the Φ13 *att*B site in strain 8325-4 (Novick, 1967) and RN4220 (Peng et al., 1988), the original Φ13 *att*B site in 8325-4 was predicted by subtracting the Φ13 sequence (accession No.: NC_004617) from the NCTC 8325 genome harboring Φ13 (accession No.: NC_007795), and aligning the resulting sequence to the known *hlb* sequence containing the Φ13 *att*B site (Coleman et al., 1991) (accession No.: X61716) for confirmation. To construct a plasmid containing a mutated Φ13 *att*B (attBmut), 800 bp upstream sequence of Φ13 *att*B site in *hlb* gene of 8325-4 (Novick, 1967) was amplified by overlapping PCR primer pairs pIMAYhlbattfor and hlbattmutrev (Supplementary Table 1), and 800 bp sequence downstream of Φ13 *att*B site was amplified by overlapping PCR primer pairs hlbattmutfor and pIMAYhlbattrev, where the Φ13 *att*B mutation from strain 61599 was included in primer hlbattmutfor (Supplementary Table 1). The PCR fragments and pIMAY vector were digested by *EcoR*I, and ligated together by Gibson Assembly® Master Mixt (BioLabs®) to form the plasmid named as pIMAY_Φ13attmut. This plasmid was electroporated into RN4220 (Kreiswirth et al., 1983) resulting in the strain RN4220pIMAY_Φ13attmut, and the Φ13 *att*B mutation was introduced into the RN4220 chromosome by homologous recombination (Monk et al., 2012). The mutation at *att*B in RN4220 was confirmed by sequencing and the strain was named RN4220Φ13attBmut.

For construction of Φ13 *att*B mutation in strain 8325-4, plasmid pIMAY_Φ13attmut was electroporated from RN4220pIMAY_Φ13attmut into 8325-4 (Novick, 1967), and the Φ13 *att*B mutation from strain 61599 was introduced to 8325-4 chromosome by homologous recombination with pIMAY allelic replacement system (Monk et al., 2012). The mutation at *att*B in 8325-4 was confirmed by sequencing and the strain was named as 8325-4Φ13attBmut.

### Determination of minimal inhibitory concentrations (MICs)

MICs of biocides and chemical compounds in strain 8325-4Φ13 were determined according to the guideline of Clinical and laboratory standards institute (Clinical and Laboratory Standards Institute (CLSI), 2008). Strains from overnight TSA plate was resuspended in 0.9% NaCl to achieve the turbidity of 0.5 McFarland standard and further diluted 100-fold in MHB. The working solution of biocides and chemical compound were benzalkonium chloride (2.67 μg/ml), hydrogen peroxide (3% w/w), Biocide 1 (5% v/v), Biocide 2 (5% v/v), Biocide 3 (5% v/v), and mitomycin C (1 μg/ml). In brief, the working solution of biocides and mitomycin C were prepared with two-fold dilution series in MHB in 96-well microtiter plates with 100 μl volume. Further, 100 μl of cell suspension was added to each well. Positive growth control of wells without biocide, and negative controls of wells with only MHB were included. The microtiter plates were incubated at 37°C for 24 h. MIC values were determined as the lowest concentration of the compounds that eliminated the visible growth of bacteria.

### Φ13 induction assay by different biocides and mitomycin C

To perform the phage induction assay, strain 8325-4Φ13 was grown to the exponential phase (OD_600_ = 0.8) (Wirtz et al., 2009) by shaking at 37°C, 200 rpm in TSB. Then, different concentration of Biocide 1, Biocide 2, Biocide 3, benzalkonium chloride, hydrogen peroxide and mitomycin C were added into the broth culture respectively, and further incubated for 2 h at 37°C, 200 rpm. The concentration series of each biocide was determined according to MIC values of strain 8325-4 Φ13 (Table 3), which included MIC, 5X MIC, 10X MIC, 20X MIC, and 30X MIC. Supernatants were sterilized by 0.45 μm pore diameter membrane filter. The phage induction levels were evaluated by PFU determination as previously described (Goerke et al., 2006b). Briefly, 100 μl of each phage supernatant dilution was mixed 100 μl indicator strain MW2c (OD_600_ = 0.1) and incubated for 10 min at room temperature before mixing with top agar and pouring onto a TSA plated with 10 μM CaCl_2_, and further incubated overnight at 37°C.

**Table 3:** MIC of strain 8325-4 for different chemical agents.

**Chemical agent**	**MIC**
Mitomycin C (mg/L)	0.125
Biocide 1 (v/v)	0.02%
Biocide 2 (v/v)	0.02%
Biocide 3 (v/v)	5.00%
Hydrogen peroxide (w/w)	0.03%
Benzalkonium chloride (mg/L)	2.67
Acetic acid (v/v)	0.16%
Amine oxide (v/v)	0.04%
Peracetic acid (v/v)	0.01%

### Lysogenization assay of Φ13 to LA-MRSA

Strain 8325-4 Φ13-kana was used as the phage donor strain. Phage lysate were obtained by growing the strain to exponential phase (OD_600_ = 0.8) at 37°C, 200 rpm, mixing with 1 μg/ml mitomycin C and further incubating for 4 h under the same condition. The culture was centrifuged at 4°C, 8,500 rpm for 6 min, and sterile filtered by 0.45 μm pore diameter membrane filter before determining the titer. Φ13 lysogens were obtained by mixing phage Φ13-kana and LA-MRSA CC398 strains (61599 and 93616) at MOIs of 0.001, 0.01, 0.1, 1, 10, 100, and 1,000 and incubating the mixture at 30°C for 30 min. Hundred microliter of the mixture was then spread on TSA plate containing 100 μl/ml kanamycin, 10 μg/ml tetracycline and 5% of sheep blood (Kan-Tet plate), and incubated at 37°C overnight. Lysogens were selected as colonies able to grow on Kan-Tet plate. The plates were stored at 4°C overnight to detect β-hemolysin activity. Lysogens were verified by colony morphology and hemolysin activity (Supplementary Figure 6) as well as by PCR to detect *ahp*A3 and *sak* genes. The lysogenization frequency was evaluated as the ratio of the Φ13 CC398 lysogen colony count (CFU/ml) on Kan-Tet plate (Φ13 CC398 lysogen) to the total recipient colony count (CFU/ml) on TSA plate with 10 μg/ml tetracycline (Tet plate).

### PCR analysis

Bacterial DNA was released by suspending 2-3 colonies in 100 μl Milli Q water and incubated at 95°C for 10 min. For confirming the transfer of Φ13 to LA-MRSA CC398 strain, primer pair sak-for/sak-rev for the *sak* gene and primer pair kanR-for/kanR-rev for the *aph* gene were used. For checking if the *hlb* gene was intact, primer pair hlbPhi13attB-for/hlbPhi13attB-rev of *hlb* gene was used. For primer sequences see Supplementary Table 1. PCR amplification was performed by mixing 12.5 μl DreamTaq Green PCR Master Mix (2X) (Thermo Fisher Scientific), 10.5 μl Milli Q water, 1 μl DNA template and 1 μl (0.1 μM) each primer. For determining the *att*B core sequence mutation in strain 8325-4 and RN4220, the PCR products of *hlb* gene were purified by GeneJet PCR purfication kit (Thermo Fisher Scientific) and sequenced by Macrogen Inc.

### *In vitro* liquid co-cultivation assay

The liquid co-cultivation assay was carried out in TSB with donor strain 8325-4Φ13-kana resistant to kanamycin and recipient strain LA-MRSA 61599 resistant to tetracycline. In brief, both donor and recipient strains were grown to exponential phase (approximately 10^8^ cfu/ml), and mixed with a ratio of 1:1. Different concentrations of hydrogen peroxide, Biocide 1 and mitomycin C were added to the culture, and then incubated at 37°C, 200 rpm for 4 h. Phage transfer was detected by plating serial dilutions on Kan-Tet plate and Tet plate followed by incubating at 37°C for 18 h. The Φ13 CC398 lysogens were counted based on the colony morphology of Φ13 lysogens (Supplementary Figure 6) and also further confirmed the PCR program of *sak* and *kan*R genes. To confirm the lysogens were belonging to CC398 strain and avoid miscounting of donor strain 8325-4Φ13-kana, a *spa*-*mec*A multiplex PCR (Tang et al., 2017) was conducted to CC398 lysogens, which could differentiate CC398 lysogens to donor strain 8325-4Φ13-kana (Supplementary Figure 5). The transfer ratio of Φ13-kana was considered as the ratio of the Φ13 CC398 lysogen colony count on Kan- Tet plates (CFU/ml) to the recipient colony count on Tet plates (CFU/ml).

## Influence of chemical agents to strains and phage

The influence of applied chemical regents to donor and recipient strains was separately evaluated by growth curve, which culture of both strains at exponential phase (approximately 10^8^ cfu/ml) was treated with a series of concentrations of mitomycin C, hydrogen peroxide and Biocide1, and measured OD_600_ value every hour till 4 h at 37°C, 200 rpm. The influence of applied chemical to Φ13-kana was evaluated by treating the phage stock with a series of concentration of chemical agents and the plaque assay was conducted after 4 h at 37°C, 200 rpm, with the plaque assay mentioned above. The influence of applied chemical regents to the induction of Φ13 with the kanamycin resistant cassette was evaluated after 4 h of treatment by Φ13 induction assay mentioned above. In addition, the influence of chemical compounds present in Biocide 1 to the donor and recipient strains was evaluated individually by growth curve and Φ13 induction assays as described above in the presence of a series concentrations of peracetic acid, amine oxide and acetic acid.

### Characterization of LA-MRSA CC398 Φ13 lysogens

Stability of the prophages in CC398 Φ13 lysogens was examined in 10 Φ13 lysogens of strain 61599 and passaging them for 20 days. In brief, 5 μl overnight culture of each lysogen was diluted 1000-fold in 5 ml TSB and incubated for 24 h at 37°C, 200 rpm. After each passage, 100 μl culture was spread on TSA plate containing 10 μg/ml tetracycline and incubated overnight at 37°C. From this plate, 50 colonies were picked up and streaked on TSA plate containing both 100 μg/ml kanamycin and 10 μg/ml tetracycline and TSA plate containing only 10 μg/ml tetracycline, and then incubated overnight at 37°C. The stability of each lysogen was determined by the ratio of colonies survived on TSA plate contain both kanamycin and tetracycline to the colonies survived on TSA plate containing only tetracycline. Further, lysogens with different Φ13 stability characteristics were sequenced by paired-end sequencing (2 × 251 bp) using Nextera XT DNA Library Preparation Kit (Illumina Inc.) on a MiSeq sequencer (Illumina Inc.). Contigs were de novo assembled using CLC-bio assembler (Qiagen).

### Lysogenization assay of Φ13 *att*B mutant

Stains 8325-4Φ13attBmut and RN4220Φ13attBmut, and their wild type strains were individually mixed with the Φ13-kana phage stock at a MOI of 0.1 and were incubated at 37°C for 4 hrs. After making serial dilutions, 100 μl of each dilution series was spread on TSA plates with 5% sheep blood containing 100 μl/ml kanamycin (Kan-plate) and TSA plate with 5% sheep blood (TSA plate), which were incubated at 37°C overnight. Lysogens were selected as colonies able to grow on Kan plate. The plates were stored at 4°C overnight to detect β-hemolysin activity. The lysogen frequency was determined as the ratio of colony count (CFU/ml) on Kan-plate to the total colony count (CFU/ml) on TSA plate. Further, PCR amplification of *hlb* gene to check if *hlb* gene was interrupted, PCR amplification of *hlb* gene including the Φ13 *att*B site was conducted for 16 colonies of lysogenized mutants.

## Results

### Induction of ΦSa3 by biocides

**Figure 1:**
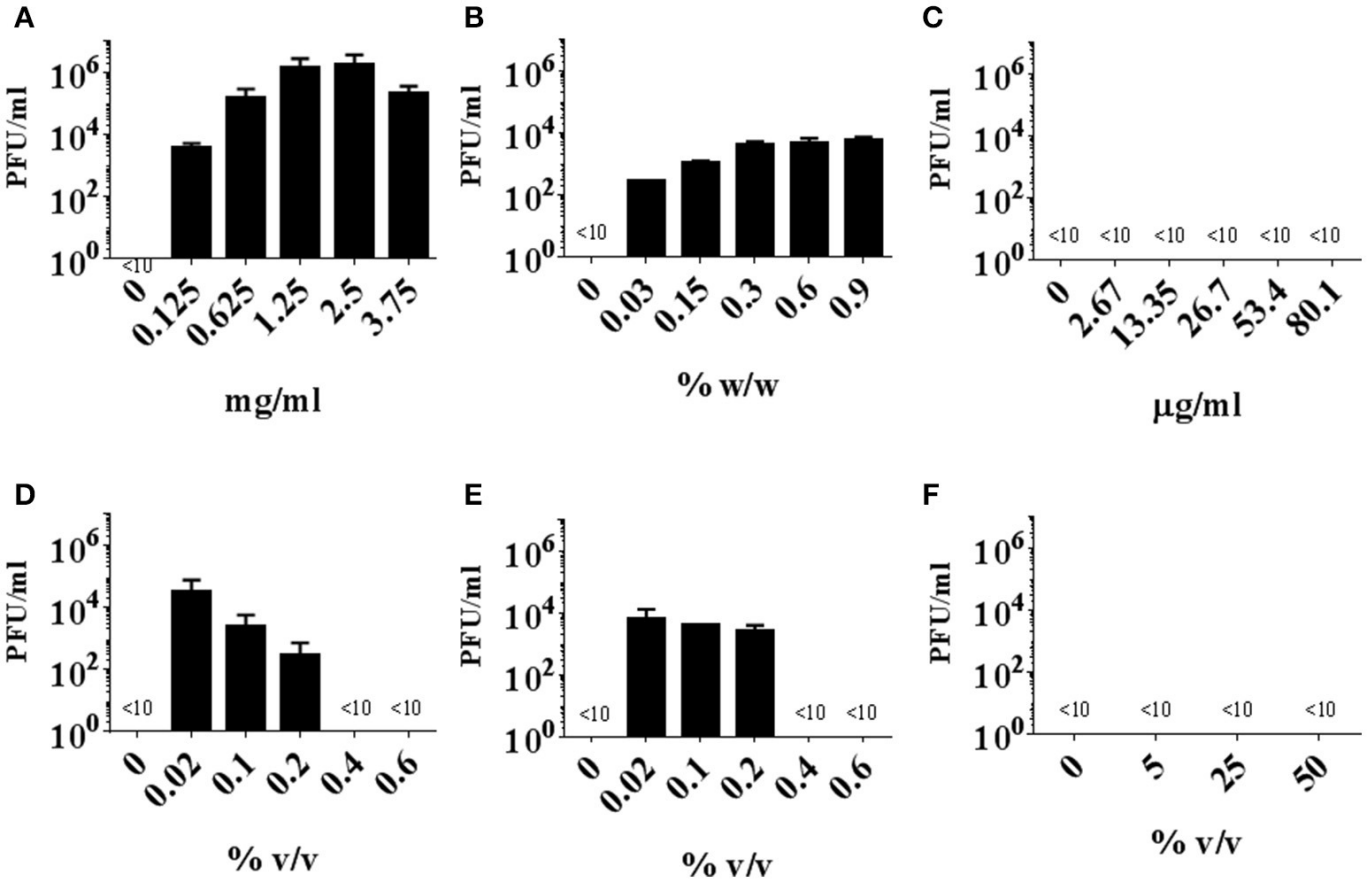
Induction of Φ13 from *S. aureus* strain 8325-4Φ13 by biocides. Strain 8325-4Φ13 was exposed to mitomycin **(A)**, hydrogen peroxide **(B)**, benzalkonium chloride **(C)**, Biocide 1 **(D)**, Biocide 2 **(E)**, Biocide 3 **(F)** at the concentrations of MIC, 5X MIC, 10X MIC, 20X MIC, and 30X MIC. The induction of Φ13 was measured by PFU counts on indicator strain MW2c, and the detection limit was more than ten PFU counts. The detection limit is < 10, the error bar represents *SD, n* = 3.

*Staphylococcus aureus* strain 8325 harbors a ΦSa3 class of phage termed Φ13 (Iandolo et al., 2002). In this study, we applied strain 8325-4Φ13 (Goerke et al., 2006a), which is a derivative of the phage cured strain 8325-4 (Novick, 1967) lysogenized with Φ13 to evaluate if commercially available biocides can induce ΦSa3. We evaluated the susceptibility of strain 8325-4Φ13 to three commercially available biocides in addition to hydrogen peroxide, benzalkonium chloride as well as mitomycin C (Table 1) by MIC (Table 3). Further, we exposed the strain 8325-4Φ13 to these biocides and chemical reagents (Table 1) and monitored plaque formation on indicator strain MW2c. Strain MW2c is a derivative of strain MW2-ΦSa3mw in which all phages have been cured (Wirtz et al., 2009). We found that excision of Φ13 was induced by mitomycin C (≤3.75 mg/ml), by biocides containing hydrogen peroxide [Biocide 1 (≤0.2% v/v) and 2 (≤ 0.2% v/v)] as well as by hydrogen peroxide (≤0.9% w/w) in a dose-dependent manner (Figures 1A–E). No induction of Φ13 was observed by benzalkonium chloride or Biocide 3 containing sodium hypochlorite as the active ingredient (Figures 1C,F) or in the absence of stimuli indicating that less than 10 pfu/ml is released.

### Transfer of Φ13 to LA-MRSA CC398

To monitor integration of Φ13 into LA-MRSA CC398, a Φ13 phage stock was obtained by mitomycin C induction from strain 8325-4Φ13-kana, a derivative of 8325-4Φ13 where the 3'-end of *chps* and *scn* in Φ13 has been replaced by the kanamycin resistance gene, *aph*A3. This phage stock was used to infect two tetracycline-resistant LA-MRSA CC398 strains 61599 and 93616 isolated from humans in Denmark (Table 2). Strain 61599 with *spa* type t034 (Larsen et al., 2015) was obtained from a pig farm worker and strain 93616 with *spa* type t899 (Larsen et al., 2016) was recovered from a mink farmer. Neither of the strains contained ΦSa3 phage by sequence analysis (Larsen et al., 2015, 2016). After repeated attempts, only strain 61599 could be lysogenised with Φ13. At multiplicity of infection (MOI) of less than one, we observed colonies resistant to both kanamycin and tetracycline, indicating lysogenization of LA-MRSA CC398 by Φ13-kana (Supplementary Figure 1). To confirm that the resulting colonies were lysogens and not spontaneous antibiotic resistant mutants, we used PCR amplification of the *aph*A3 and *sak* genes of Φ13-kana (data not shown).

To examine if Φ13-kana could transfer directly from *S. aureus* 8325-4Φ13-kana (donor strain) to recipient LA-MRSA CC398 strain 61599 in the presence of biocides or mitomycin, we first assessed a series of concentrations of mitomycin C, hydrogen peroxide and Biocide 1 ranging from sub-lethal to lethal with respect to their influence on growth (Supplementary Figure 2) and the induction levels of Φ13-kana (Supplementary Figure 3). We also confirmed that these concentrations have minimum effect on Φ13-kana in plaque assays (Supplementary Figure 4). Further, we evaluated MIC of strain 8325-4Φ13 to acetic acid, amine oxide and peracetic acid (Table 1), which are present in Biocide 1 (Table 3), and exposed the strain to these chemicals to evaluate their effect on growth and Φ13 induction (Supplementary Figure 5). We found that, in addition to hydrogen peroxide (Supplementary Figure 3B), Φ13-kana can also be induced by peracetic acid, which is able to spontaneously decompose to hydrogen peroxide and acetic acid (Yuan et al., 1997; Supplementary Figure 6).

**Figure 2:**
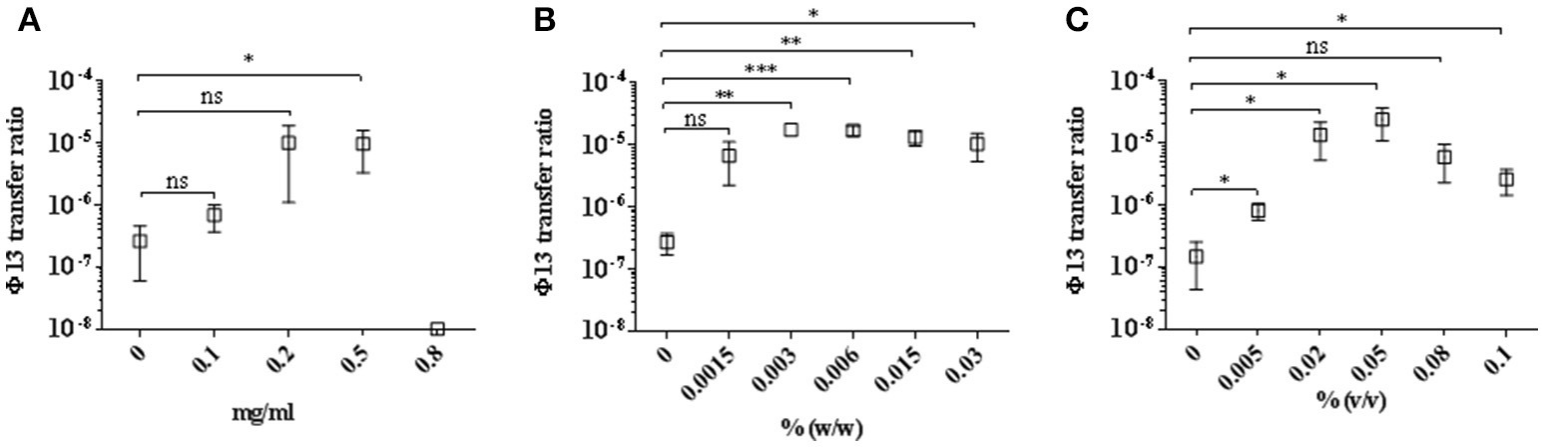
Transfer of Φ13 during co-cultivation of LA-MRSA CC398 with 8325-4Φ13-kana. Strains LA-MRSA CC398 and 8325-4Φ13-kana were co-cultivated and exposed for 4 h to different concentrations of **(A)** mitomycin C, **(B)** hydrogen peroxide and **(C)** Biocide 1. Open squares represent the Φ13 transfer ratio calculated as the number of colonies resistant to tetracycline and kanamycin relative to the number resistant to tetracycline. Error bars represent ±*SD, n* = 3. ^***^P < 0.001, ^**^P < 0.01 and ^*^P < 0.05, NS, none significant by *t*-test analysis.

We co-cultured the donor strain 8325-4Φ13-kana with the recipient strain 61599, in the presence of sub-lethal to lethal concentrations of mitomycin C, hydrogen peroxide, and Biocide 1. Colonies of strain 61599 lysogenized with Φ13-kana were detected by plating on blood plates containing kanamycin and tetracycline. The transfer of Φ13-kana was confirmed by PCR for *sak* and *aph*A3 and the identity of the resulting colonies was confirmed by PCR amplification of *spa* and *mec*A (Supplementary Figure 7). We observed that Φ13-kana transferred from strain 8325-4Φ13-kana to 61599 with a transfer frequency between 10^−5^ and 10^−6^ in the presence of 0.002–0.1%(v/v) Biocide 1, which contains hydrogen peroxide as the active compound (Figure 2C). Exposure to mitomycin C and hydrogen peroxide also induced the transfer of Φ13-kana (Figures 2A,B). Surprisingly, in the absence of DNA damaging agents, we also observed a transfer frequency of Φ13-kana was around 2.3 × 10^−7^ (Figure 2). These results show that sublethal to lethal concentrations of biocides containing hydrogen peroxide and mitomycin C promote transfer of Φ13 between human and livestock-adapted strains *in vitro*.

### Characterization of Φ13 in CC398 lysogens

**Figure 3:**
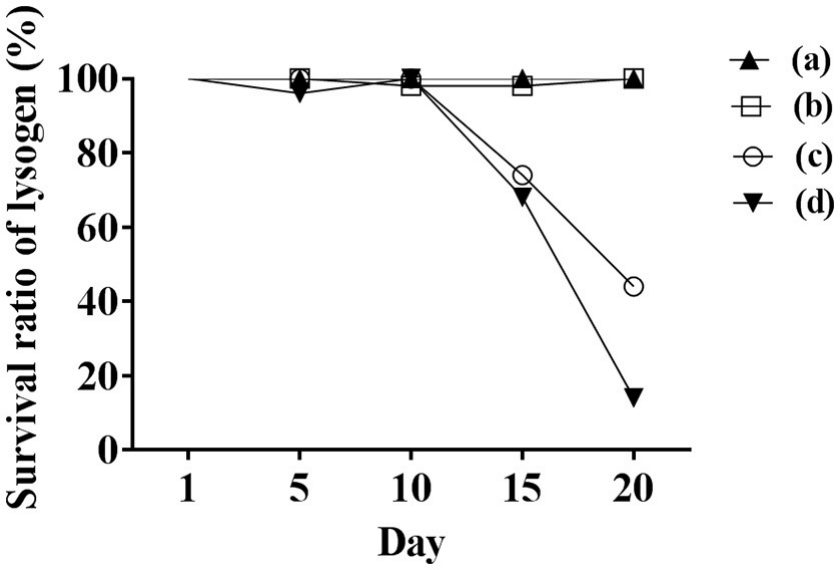
Φ13 stability in LA-MRSA CC398 strain 61599. Nine lysogens were grown over a 20 period with 1000 fold dilution performed daily from the previous day overnight culture. The stability of the phage was monitored by plating on agar plates with tetracycline, and scoring the number that were also kanamycin resistant. (a) filled triangle represents LY01-LY06 of lysogens, which were fully stable with Φ13 prophages during the 20-day test; (b) opened square represents LY07, which had sporadically lost Φ13 during 20 days; (c) open circle represent LY08 and (d) filled inverted triangle represents LY09, both lost Φ13 in the majority of the analyzed colonies after 20 days, respectively.

Initially, we assessed hemolysin production of nine Φ13 lysogens (LY01-09) in LA-MRSA CC398 strain 61599 on blood agar plates and observed that all were β-hemolysin positive (Supplementary Figure 8) suggesting that the phage had not integrated in the *hlb* attachment site commonly preferred in human strains (Goerke et al., 2006b). Subsequently, we evaluated stability of the lysogens over a period of 20 days (corresponding to approximately 960 generations) by plating on tetracycline agar plates and recording the fraction of kanamycin-resistant colonies (Figure 3). Here we observed different stability patterns with Φ13-kana being completely stabile in six lysogens (LY01-06), and being partially stable in the rest of the lysogens with 44% (LY08) and 29% (LY09) of cells retaining the phage, respectively. In LY07, the percentage of cells carrying the phage varied between 96 and 100% during the experiment indicating sporadic loss. To examine the genetic basis for the different stability patterns we sequenced the genomes of lysogens and observed eight different Φ13 integration sites in strain 61599 (Table 4). In the six lysogens (LY01-LY06) where Φ13-kana was completely stable, the phage was integrated at six different locations in the chromosome of 61599. In five of the six lysogens (LY01-05), Φ13-kana was inserted in annotated genes, whereas in LY06 the phage was integrated in an unrelated intergenic region (Table 4). In LY07, where Φ13-kana stability varied the phage was integrated in a gene encoding a hypothetical protein located on plasmid JQ861959 that had integrated in the chromosome, which may explain why recovery of the phage varied from plating to plating. In both LY08 and LY09 from which the phage was lost in the majority of cells after the 20 day period, Φ13-kana was integrated in *yozB* encoding a putative membrane protein, the function of which is unclear. These results strongly indicate that Φ13 integrates at alternative sites in LA-MRSA CC398 and that the overall stability of the integrated phage is influenced by its integration site in the chromosome.

**Table 4:** Φ13 integration site in LA-MRSA CC398 strain 61599.

**Lysogen No**.	Φ13 integration sites^*^	**Function of gene integrated by Φ13**	*att*B core sequence (5′ to 3′)^**^
LY01	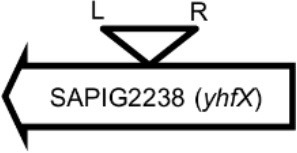	Alanine racemase	GTTATCCAATCTGG
LY02	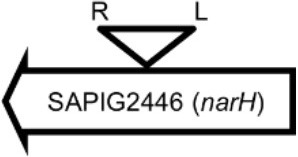	Nitrate reductase	GGGGACCTAACTGG
LY03	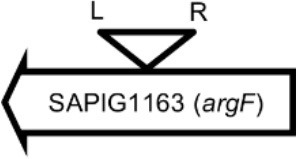	Ornithine carbamoyltransferase	CCATTCCATACTGG
LY04	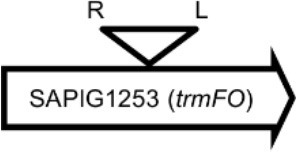	FADH(2)-oxidizing methylenetetrahydrofolate–tRNA-(uracil(54)-C(5))-methyltransferase TrmFO)	GTGTATCCATCTGG
LY05	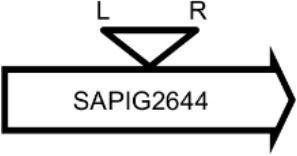	Acyl esterase	TTTATCGTTTCTGG
LY06	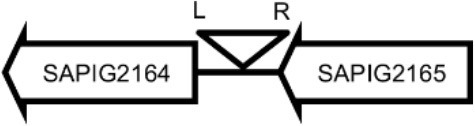	SAPIG2164: aldehyde dehydrogenase family protein SAPIG2165: HxlR family transcriptional regulator	TTTATCCGTAATGC
LY07	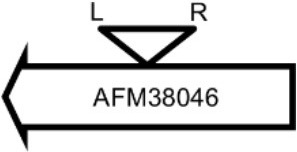	Plamid JQ861959 integrated upstream of the *mut*B gene	*TGT*TCTTT*A*T*CTGG*
LY08	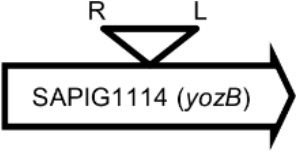	Membrane protein	GTTTCTCCACCTGG
LY09	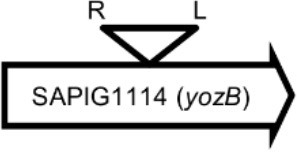	Membrane protein	GTTTCTCCACCTGG
*S. aureus* COL	*att*B in *hlb* gene	Φ13 attachment site in *hlb* described by Coleman et al. (1991)	TGTATCCAAACTGG

* *Φ13 integration site is indicated by open, inverted triangles; open arrows represent ORFs with gene name and accession number. Letter L denotes the position of attL, and letter R denotes the position of attR*.

** *Letters with underline represent the nucleotides in attB that correspond to the core sequence of attB in hlb described by Coleman et al. (1991)*.

**Figure 4:**
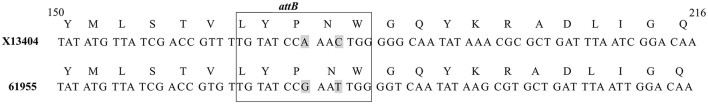
Core sequence of attachment site (*att*B) [boxed] in *hlb* gene in *S. aureus* COL (Accession No. X13404) compared to *att*B in the *hlb* gene of strain 61599. Two point mutations occurred in *att*B of *hlb* gene in strain 61599. The selected nucleotide sequence was part of the X13404 CDS region (CAA31769). The gray shaded letters represent the variations in the *attB* of strain 61599.

**Figure 5:**
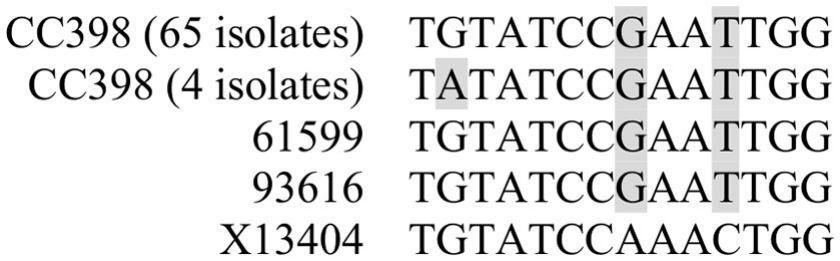
Variation of *att*B core sequence for Φ13 in *hlb* gene in ΦSa3-negative CC398 isolates compared to the *att*B in *hlb* gene of *S. aureus* COL (accession no. X13404) described by Coleman et al. (1991). CC398 isolate information was obtained from the study of Price et al. (2012). The gray shaded letters represent the variations in the *att*B of CC398 isolates.

By sequence analysis, we found that all integration sites in the nine lysogens were partially similar to the *att*B sequence for Φ13 in *hlb* gene originally described by Coleman et al. (1991) (Table 4). However, we were not able to observe other similarities between the integration sites or regions. By further comparing these integration sites in lysogens to the corresponding sequences in strain 61599 and strain 8325-4, we found that only the integration site in LY03 contained one nucleotide variation between the strain 61599 and strain 8325-4, but this variation did not enhance resemblance to the *att*B in the *hlb* gene (Supplementary Table 2). In addition, sequence analysis of the *hlb* gene of strain 61599 revealed variation at two residues in the 14 nucleotide *att*B sequence when compared to *att*B in *hlb* gene of the human derived strain *S. aureus* COL (Projan et al., 1989). Both nucleotide substitutions are silent leaving the β-hemolysin expression intact (Figure 4). This finding suggests that point mutations in *hlb* of LA-MRSA may drive Φ13 to integrate elsewhere in the chromosome. To test this hypothesis, we compared the *att*B core sequence of ΦSa3 from strain 61599 to the 69 of genome sequenced CC398 isolates from a previous study by Price et al. (2012). We observed that 65 out of 69 CC398 isolates from Price et al.'s study showed the same substitutions of the *att*B core sequence for Φ13 in the *hlb* gene as we found in strain 61599, while four of CC398 isolates had three substitutions compared to the *att*B core sequence in *hlb* described by Coleman et al. (1991; Figure 5).

**Figure 6:**
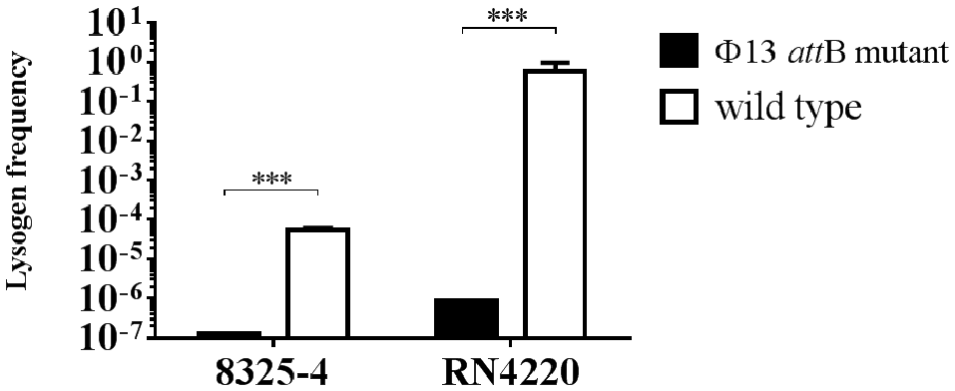
Lysogenization of 8325-4Φ13attBmut and RN4220Φ13attBmut by Φ13. Φ13-kana were mixed with 8325-4Φ13attBmut, RN4220Φ13attBmut, and the corresponding wild type strains with MOI of 0.1 and lysogen frequency was monitored as ratio of CFU on TSA plates with 100 μg/ml kanamycin and 5% of sheep blood (Φ13-kana LA-MRSA CC398 lysogen) relative to the total CFU count on TSA plates with 5% of sheep blood. Horizontal lines represent mean value, error bar represents ±*SD, n* = 3. ^***^P < 0.001 by *t*-test analysis.

To support our hypothesis that the mutation at *att*B in *hlb* of CC398 isolates influences integration of ΦSa3, we mutated the Φ13 *att*B site of *hlb* both in strain 8325-4 deviated from NCTC 8325 cured of Φ11, Φ12, and Φ13 (Novick, 1967), and in strain RN4220 derived from 8325-4 (Peng et al., 1988). After infecting both *att*B mutated strains with Φ13-kana at MOI of 0.1, we observed a significant decrease in lysogenization of Φ13-kana in both *att*B mutant strains, when compared to the respective wild type control strains (Figure 6) and confirmed by PCR amplification of *hlb* (Supplementary Figure 9) and *aph*3 (Supplementary Figure 10). These results demonstrate that point mutations of *att*B core sequence in *hlb* of CC398 isolates strongly influence the integration of ΦSa3 into the *hlb* gene, and the mutation favors ΦSa3 to integrate elsewhere in the bacterial genome and keeps the *hlb* intact.

## Discussion

Mitomycin C and hydrogen peroxide are known to induce prophages in bacteria by causing DNA damage, which activates the SOS response and in turn cleaves the phage repressor (Frye et al., 2005; Goerke et al., 2006a; Loś et al., 2010; Nanda et al., 2015). Here, we show that commercial biocides containing hydrogen peroxide can induce phage Φ13, a phage belonging to Sa3 phage group that is known to encode human immune evasion genes and likely to be important for human colonization (Iandolo et al., 2002). Additionally, when co-culturing the human originating strain, 8325-4Φ13-kana with the livestock MRSA CC398 strain 61599 (*spa* type t034), the transfer frequency of Φ13 to strain 61599 was significantly increased in the presence of sublethal concentrations of hydrogen peroxide or commercial biocides containing hydrogen peroxide when compared to the transfer frequency without any treatment. Integration of Φ13 was also attempted in another LA-MRSA CC398 strain namely 93616 (*spa* type t899), but in this strain, we did not obtain lysogens. The inability of Φ13 to lysogenize strain 93616 could be caused by strain variations such as the restriction modification systems that restricts DNA transfer between staphylococcal lineages (Sadykov, 2016), or differences in wall teichoic acid glycopolymers that are the receptors of Φ13 and are highly strain-specific (Xia et al., 2010).

Hydrogen peroxide is a commonly applied bactericidal compound for disinfection in livestock production (Kjølholt et al., 2001). Our study indicates that this compound can act as an extrinsic factor contributing to the induction of ΦSa3 by triggering excision and propagation of the prophage, and subsequent transfer from human to livestock-associated *S. aureus* strains. In contrast, neither sodium hypochlorite that is known to affect the bacterial cell wall (Maillard, 2002), nor benzalkonium chloride that is a membrane-active agent (SCHENIHR (Scientific Committee on Emerging and Newly Identified Health Risks), 2009), were able to induce Φ13. In the absence of external stimuli, no spontaneous induction of Φ13 was observed in concordance with a previous study (Goerke et al., 2006a). Interestingly, we did observe transfer of Φ13 from strain 8325-4Φ13-kana to strain 61599 when the two strains were co-cultured. The most likely reason for this is that Φ13 propagates on strain 61599 resulting in more phages that in turn increase the chance of phage integration in strain 61599.

ΦSa3 group is known as *hlb*-converting phages that integrate in the *hlb*-gene at the *att*B attachment site (5′-TGTATCCAAACTGG-3′) recognized by the phage integrase (Coleman et al., 1991). A likely explanation for the atypical integration of ΦSa3 in strain 61599 is the two silent point mutations in the *att*B core sequence located within *hlb*. We speculate that these mutations in *att*B may be the reason that we did not observe phage integration in *hlb* but rather at numerous other locations in the LA-MRSA CC398 strain 61599. These integration sites share homology to the *att*B core sequence for Φ13 (Coleman et al., 1991). In particularly seven of the eight integration site sequences contain a four nucleotides sequence (5′-CTGG-3′) at the 3′-end, which is present in the Φ13 *att*B core sequence in *hlb* gene (5′-TGTATCCAAACTGG-3′) (Coleman et al., 1991), but is not in the *att*B core sequence in *hlb* from CC398 strains (5′-TGTATCCGAATTGG-3′ and 5′-TATATCCGAATTGG-3′). We speculate that this sequence similarity is important for the integration of ΦSa3 into CC398. It was previously reported that Φ13 can be integrated at different locations in *S. aureus* (Goerke et al., 2006b; Kraushaar et al., 2017), but the reason for the atypical integration of ΦSa3 had not been studied before. Here, we exchanged the core sequence of Φ13 *att*B in *hlb* from both strain 8325-4 and strain RN4220 to the *att*B core sequence from CC398 strain 61599. After infecting with Φ13, we observed a significant decrease in lysogenization frequency in the Φ13 *att*B mutants compared to cells carrying the intact *hlb* gene. This result demonstrates that the mutations at the *att*B core sequence in *hlb* gene of CC398 strain cause the low transfer frequency of Φ13 to CC398 strains and also drives the atypical integration of Φ13 in CC398 strains.

In conclusion, we show that in the presence of commercially available biocides containing hydrogen peroxide, ΦSa3 is transferred from a human-associated *S. aureus* donor strain to a strain, belonging to the CC398 complex. Previous studies have shown that the CC398 ancestor was adapted to humans but jumped to animals by loss of prophage Sa3 during the close human-livestock activities (Price et al., 2012). Recently, it was shown that ΦSa3 may be re-introduced into livestock-associated CC398 strains in a single horizontal gene transfer event from human-associated *S. aureus* and that it can be maintained stably as a prophage in the CC398 lysogens (Larsen et al., 2016). Our study highlights the importance of environmental factors in transfer of ΦSa3 between *S. aureus* strains and that just a four nucleotides sequence may be enough to guide integration of the phage LA-MRSA strains. Future studies will be needed to determine if and how integration frequencies vary from strain to strain and to identify factors and conditions that may prevent ΦSa3 transmission.

## Author contributions

YT, JH, LN, CWo, and HI designed the study, YT and AH conducted the microbiological analysis, CWi and YT constructed strains, YT, JL, JH and HI analyzed the results. YT wrote the manuscript and YT, LN, AH, JH, CWi, PA, JL, CWo, and HI reviewed the manuscript.

### Conflict of interest statement

The authors declare that the research was conducted in the absence of any commercial or financial relationships that could be construed as a potential conflict of interest.
