# Supplementary material for: Commercial Biocides Induce Transfer of Prophage Φ13 from Human Strains of Staphylococcus aureus to Livestock CC398
Source: Front Microbiol. 2017 Dec 7;8:2418. doi: 10.3389/fmicb.2017.02418 (PMC5726172; doi:10.3389/fmicb.2017.02418)
Supplement: Supplementary file 1 [file DataSheet1.PDF]

Supplementary Material

Commercial biocides induce transfer of prophage Φ13 from *Staphylococcus aureus* human strain to livestock CC398

Yuanyue Tang[1], Lene Nørby Nielsen[2], Annemette Hvitved[2], Jakob Krause Haaber[3], Christiane Wirtz[4], Paal Skytt Andersen [1,5], Jesper Larsen[5], Christiane Wolz[4], Hanne Ingmer[1,*]

[1] Department of Veterinary and Animal Science, Faculty of Health and Medical Sciences, University of Copenhagen, Frederiksberg C, Denmark

[2] National Food Institute, Technical University of Denmark, Søborg, Denmark

[3] DTU Biosustain, Technical University of Denmark, Kgs. Lyngby, Denmark

[4] Institut für Medizinische Mikrobiologie und Hygiene, Universitätsklinikum Tübingen, Tübingen, Germany

[5] Department of Microbiology and Infection Control, Statens Serum Institut, Copenhagen S, Denmark

***Correspondence:** Hanne Ingmer: hi@sund.ku.dk

1 Supplementary Figures and Tables

1.1 Supplementary Tables

Supplementary Table 1. Primers used in this study

Primer	Sequence	Description	Reference
**hlbattmutrev**	atattgcccCCAATTCGGATACAAAACGGTCG	Overlapping PCR for pIMAY_Φ13attmut, 800 bp	This study
**pIMAYhlbattfor**	tcgataagcttgatatcgTAATATTTTGATTCTTATTAAATGTTAAGCT		This study
**hlbattmutfor**	ccgaattggGGGCAATATAAACGCGCTGA	Overlapping PCR, for pIMAY_Φ13attmut, 800 bp	This study
**pIMAYhlbattrev**	gatcccccgggctgcaggTAGGCTTTGATTGGGTAATGATCTG		This study
**hlbPhi13attB-for**	AGGAGTGATAATGATGGTGA	*hlb* gene flanking include Φ13 *att*B core sequence, 566bp	This study
**hlbPhi13attB-rev**	GTGTATGTGTACCGATAACG		This study
***kan*R-for**	CCGAGGTATGAAAACGAGAATTGG	*aph*A3, kanamycin resistant cassatte, 1418bp	This study
***kan*R-rev**	GGGACCCCTATCTAGCGAACTTT		This study
***sak*-for**	GTGCATCAAGTTCATTCGAC	*sak*, encoding staphylokinase, 384 bp	This study
***sak*-rev**	TAAGTTGAATCCAGGGTTTT		This study

**spa-1113f**	TAAAGACGATCCTTCGGTGAGC	*spa,* encoding protein A;	(Tang et al., 2017)
**spa-1514r**	CAGCAGTAGTGCCGTTTTGCTT		(Tang et al., 2017)
**mecA P4**	TCCAGATTACAACTTCACCAGG	*mec*A, methicillin resistance;	(Tang et al., 2017)
**mecA P7**	CCACTTCATATCTTGTAACG		(Tang et al., 2017)

Supplementary Table 2. The alignment of Φ13 integration site in different genes among lysogens, strain 61599 and strain 8325-4*

Lysogen No.	Integration site aligment**	
LY01	LY01	GTTATCCAATCTGG
	61599	GTTATCCAATCTGG
	8325-4	GTTATCCAATCTGG
LY02	LY02	GGGGACCTAACTGG
	61599	GGGGACCTAACTGG
	8325-4	GGGGACCTAACTGG
LY03	LY03	CCATTCCATACTGG
	61599	CCATTCCATACTGG
	8325-4	CCATTCCACACTGG
LY04	LY04	GTGTATCCATCTGG
	61599	GTGTATCCATCTGG
	8325-4	GTGTATCCATCTGG
LY05	LY05	TTTATCGTTTCTGG
	61599	TTTATCGTTTCTGG
	8325-4	TTTATCGTTTCTGG
LY06	LY06	TTTATCCGTAATGC
	61599	TTTATCCGTAATGC
	8325-4	TTTATCCGTAATGC
LY07	LY07	TGTTCTTTATCTGG
	61599	TGTTCTTTATCTGG
	8325-4	TGTTCTTTATCTGG
LY08	LY08	GTTTCTCCACCTGG
	61599	GTTTCTCCACCTGG
	8325-4	GTTTCTCCACCTGG
LY09	LY09	GTTTCTCCACCTGG
	61599	GTTTCTCCACCTGG
	8325-4	GTTTCTCCACCTGG
att*B in hlb**		**TGTATCCAAACTGG**

*The sequence of strain 8325-4 was derived from NCTC8325 (accession no. NC_007795) with manually deletion of Φ11, Φ12, Φ13.

** The grey shaded letters represent the nucleotide variation in integration site of lysogens and strain 61599 when compared to 8325-4

*** The *att*B in *hlb* gene is from Coleman et al. (1991) in *S. aureus* COL (accession no. X13404).

1.2 Supplementary Figures

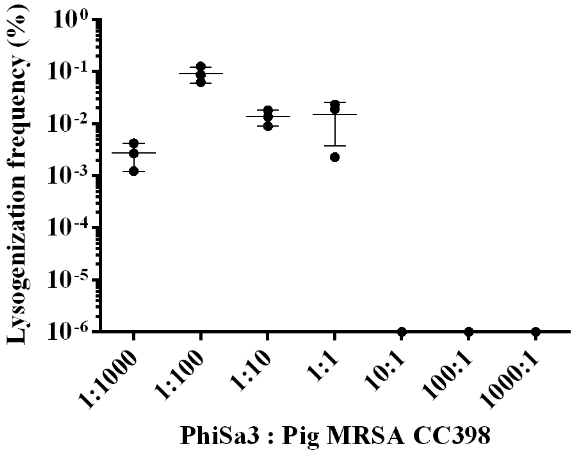

Supplementary Figure 1. Lysogenization of LA-MRSA CC398 strain 61599 by Φ13. Φ13-kana and strain 61599 were mixed in various ratios and integration frequency was monitored as CFU on TSA plates with 100 µg/ml kanamycin, 10 µg/ml tetracycline and 5% of sheep blood (Φ13-kana LA-MRSA CC398 lysogen) relative to the total CFU count on TSA plates with 10 µg/ml tetracycline (CC398 recipient). The filled circle represents the lysogenization frequency of three independent experiments and horizontal lines represents mean value, error bar represents ±SD, n=3.

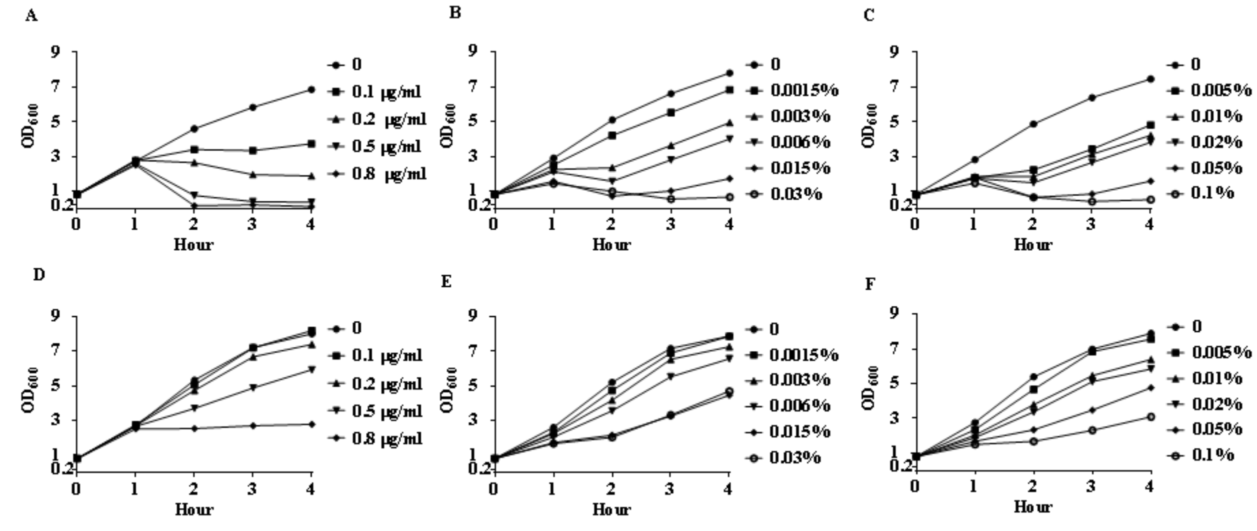

Supplementary Figure 2. Growth curves for LA-MRSA CC398 strain 61599 and strain 8325-4Φ13kana in the presence of mitomycin C or biocides. LA-MRSA CC398 strain 61599 (**A, B, C**) and strain 8325-4Φ13-kana (**D, E, F**) were treated with different concentration of mitomycin C (**A,D**), hydrogen peroxide and (**B, E**) or Biocide 1 (**C, F**). The growth was monitored at OD = 600nm.

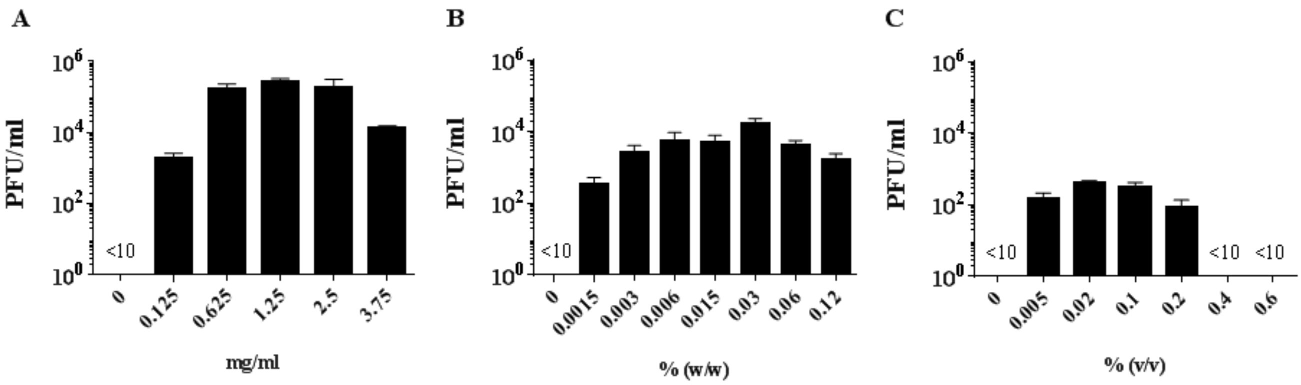

Supplementary Figure 3. Induction of Φ13-kana from strain 8325-4Φ13-kana. Strain 8325-4Φ13-kana was treated for 4 hrs with different concentrations of mitomycin C (**A**), hydrogen peroxide (**B**) and Biocide 1 (**C**). PFUs of Φ13-kana were monitored on indicator strain MW2c. Error bars represent ±SD, n=3.

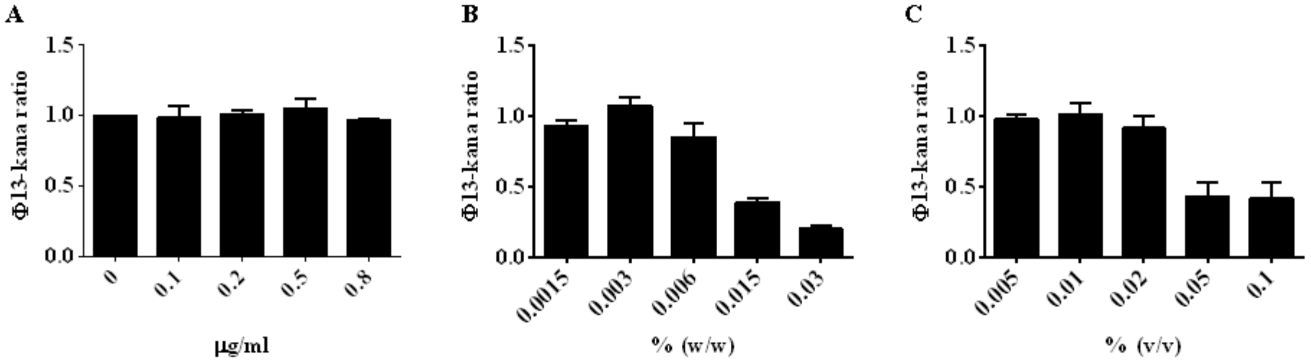

Supplementary Figure 4. Stability of Φ13-kana in biocides and mitomycin C. Φ13-kana was exposed to different concentrations of mitomycin C (**A**), hydrogen peroxide (**B**) and Biocide 1 (**C**) for 4 hours. The stability of Φ13-kana was evaluated as PFU on indicator strain MW2c, and normalized to the number of PFU in the absence of exposure. Error bars represent ±SD, n=3.

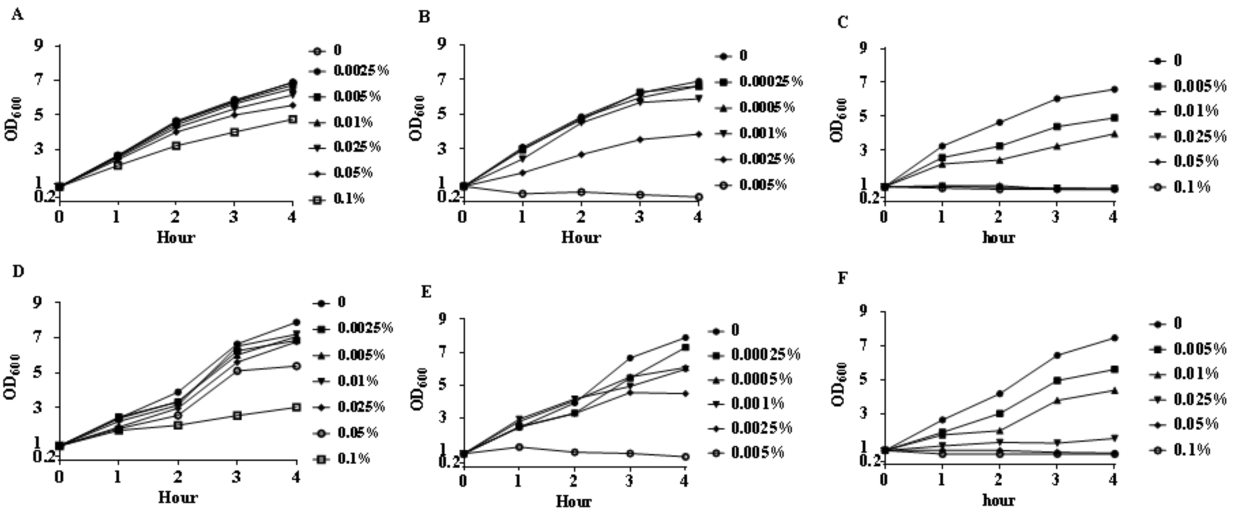

Supplementary Figure 5. Growth curves for LA-MRSA CC398 strain 61599 and strain 8325-4Φ13kana in the presence of acetic acid, amine oxide and peracetic acid. LA-MRSA CC398 strain 61599 (**A, B, C**) and strain 8325-4Φ13-kana (**D, E, F**) were treated with different concentration of acetic acid (**A,D**), amine oxide (**B, E**) or peracetic acid (**C, F**). The growth was monitored at an optical density at 600nm.

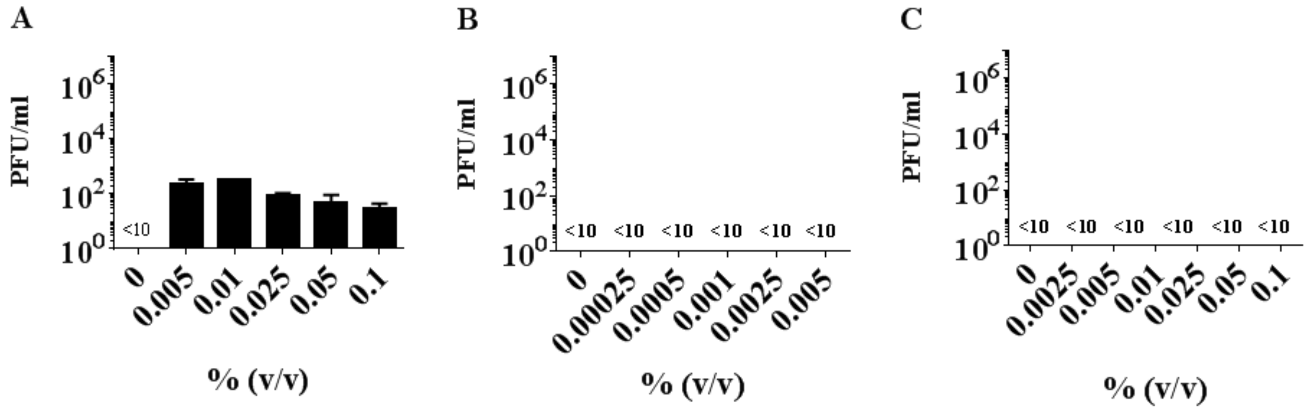

Supplementary Figure 6. Induction of Φ13-kana from strain 8325-4Φ13-kana. Strain 8325-4Φ13-kana was treated for 4 hrs with different concentrations of peracetic acid (**A**), acetic acid (**B**) or amine oxide (**C**). PFUs of Φ13-kana were monitored on indicator strain MW2c. Error bars represent ±SD, n=3.

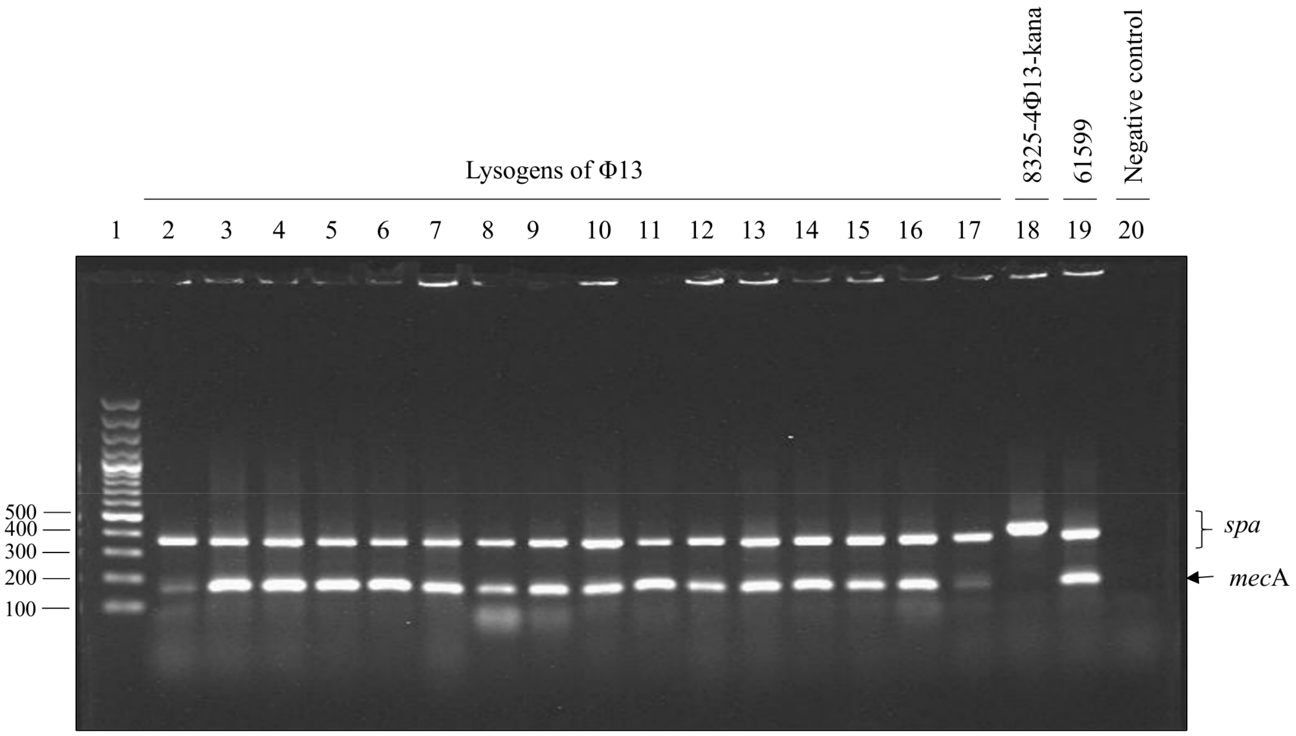

Supplementary Figure 7. Verification of LA-MRSA CC398 Φ13 lysogens by *spa*-*mec*A multiplex PCR. Lanes 2 to 17 represent potential lysogens randomly picked single colonies that grew on Tet-Kan plates from co-cultivation of strain 8325-4Φ13-kana (lane 18) and strain 61599 (lane 19). Lane 1 was GeneRuler 100 bp Plus DNA Ladder (Thermo Scientific), and lane 20 was the negative control without DNA template. Multiplex PCR products were separated on a 1% agarose gel and stained with ethidium bromide.

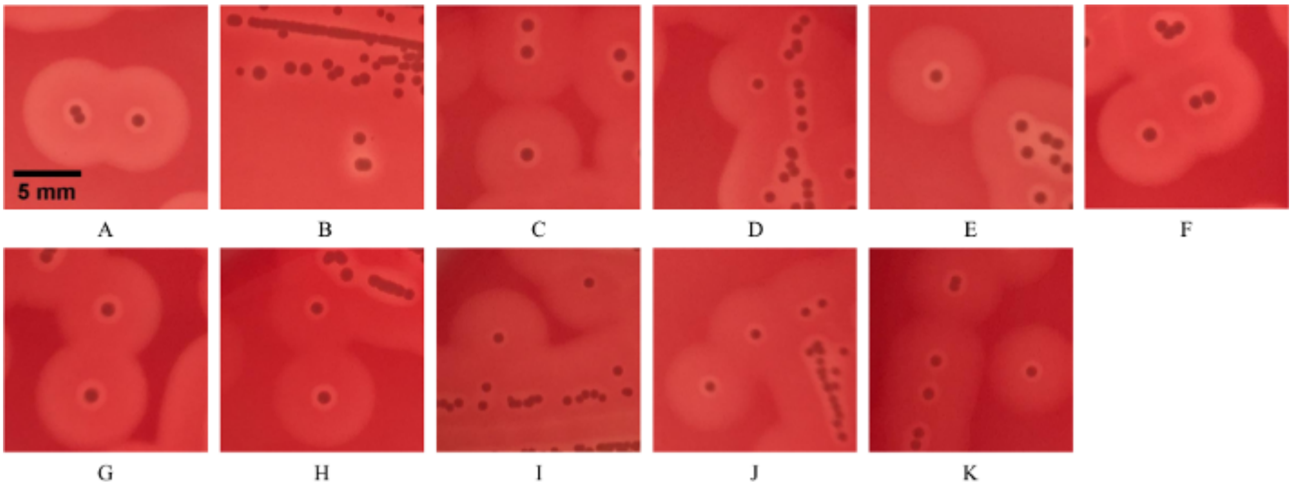

Supplementary Figure 8. Hemolytic activity of LY01-09. β-hemolysin activity was assessed on blood agar plates with 5% sheep blood after 18 hours incubation at 37 °C and subsequent storage at 4 °C overnight of strain 61599 (**A**), strain 8325-4-Φ13-kana (**B**) and lysogens LY01- LY09 (**C-K**).

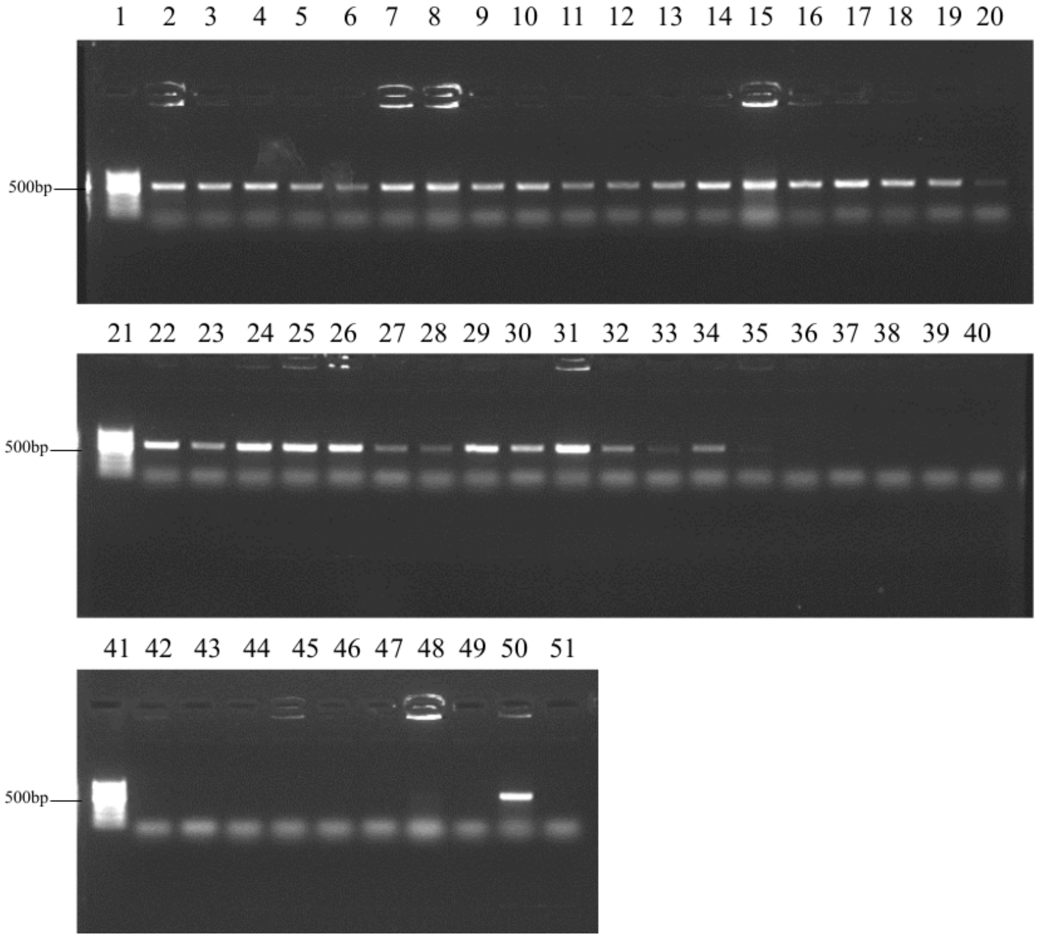

Supplementary Figure 9. Verification of Φ13 lysogens by PCR amplification of *hlb*. PCR using the primer pair hlbPhi13attB-for/hlbPhi13attB-rev was used to assess lysogeny. Lanes 2 to 17 represent potential lysogens from Φ13 infection of strain 8325-4Φ13attBmut; Lanes 18, 19 and 22 to 27 represent potential lysogens from Φ13 infection of strain RN4220Φ13attBmut. The positive PCR results indicated the intact of *hlb* gene in lysogens of *att*B mutants. Lanes 36 to 40, 42 and 43 represent potential lysogens from Φ13 infection of strain 8325-4; Lanes 44 to 49 represent potential lysogens from Φ13 infection of RN4220. The negative results indicated the disruption of *hlb* gene due to the Φ13 integration. Lanes 1, 21 and 41 are GeneRuler 1kp Plus DNA Ladder (Thermo Scientific), and lane 50 is the positive control using strain RN4220 and Lane 51 is the negative control lacking DNA template. PCR products were separated on a 1% agarose gel and stained with ethidium bromide.

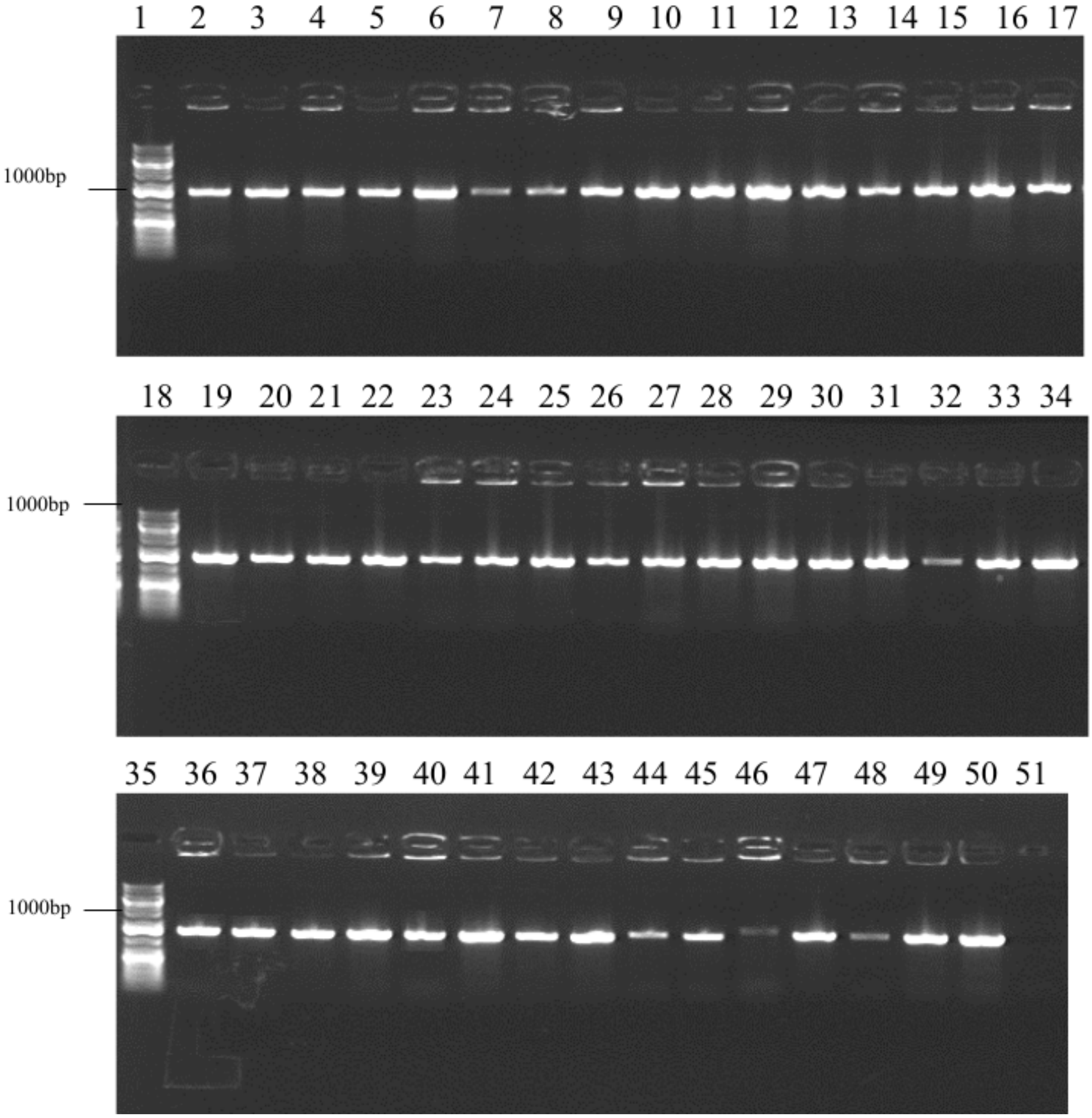

Supplementary Figure 10. Verification of Φ13 lysogens by PCR amplification of *aph*A3 kanamycin resistance gene. PCR using primers primer pair Kan-for/Kan-rev were used to assess lysogeny. Lanes 2 to 17 are potential lysogens from Φ13 infection of strain 8325-4Φ13attBmut; Lanes 19 to 34 are potential lysogens from Φ13 infection of strain RN4220Φ13attBmut. Lanes 36 to 41 are potential lysogens from Φ13 infection assay of strain 8325-4; Lanes 42 to 49 are potential lysogens from Φ13 infection of RN4220. Lanes 1, 18 and 35 are GeneRuler 1kp Plus DNA Ladder (Thermo Scientific), and lane 50 is the positive control using 8325-4Φ13-kana DNA and Lane 51 is the negative control lacking DNA template. PCR products were separated on a 1% agarose gel and stained with ethidium bromide.

2 Reference

Coleman, D., Knights, J., Russell, R., Shanley, D., Birkbeck, T.H., Dougan, G., and Charles, I. (1991). Insertional inactivation of the *Staphylococcus aureus* beta-toxin by bacteriophage phi 13 occurs by site- and orientation-specific integration of the phi 13 genome. *Mol Microbiol* **5:** 933-939

Tang, Y., Larsen, J., Kjeldgaard, J., Andersen, P.S., Skov, R., and Ingmer, H. (2017). Methicillin-resistant and -susceptible *Staphylococcus aureus* from retail meat in Denmark. *Int J Food Microbiol* **249:** 72-76. doi: 10.1016/j.ijfoodmicro.2017.03.001
